# Supplementary material for: Enhanced Electroluminescence from a Silicon Nanocrystal/Silicon Carbide Multilayer Light-Emitting Diode
Source: Nanomaterials (Basel). 2023 Mar 20;13(6):1109. doi: 10.3390/nano13061109 (PMC10051929; doi:10.3390/nano13061109)
Supplement: Supplementary file 1 [file nanomaterials-13-01109-s001.zip › nanomaterials-2268180-supplementary.pdf]

nanomaterials

Supplementary Materials

Enhanced Electroluminescence from a Silicon Nanocrystal/Silicon Carbide Multilayer Light-Emitting Diode

Teng Sun [1], Dongke Li [1,2], Jiaming Chen [1], Yuhao Wang [1], Junnan Han [1], Ting Zhu [1], Wei Li [1], Jun Xu [1,*] and Kunji Chen [1]

1 School of Electrical Science and Engineering, Collaborative Innovation Centre of Advanced Microstructures, Jiangsu Provincial Key Laboratory of Advanced Photonic and Electrical Materials, Nanjing University, Nanjing 210000, China
2 ZJU-Hangzhou Global Scientific and Technological Innovation Centre, School of Materials Science and Engineering, Zhejiang University, Hangzhou 311200, China
* Correspondence: author: junxu@nju.edu.cn

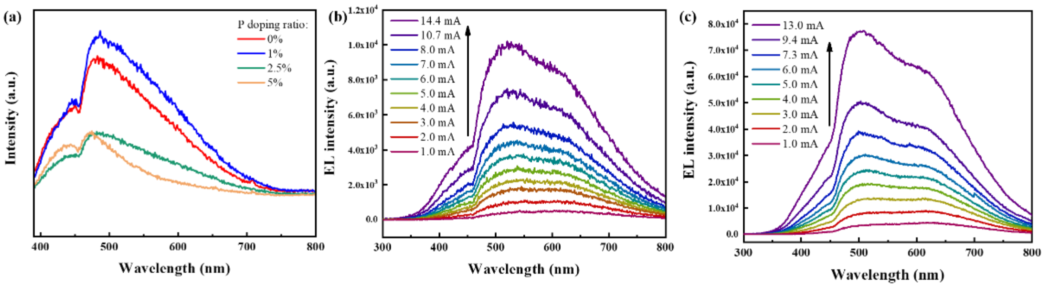

Figure S1. Original detected spectra of (**a**) PL spectra with various doping ratios, (**b**) EL spectra of un-doped Si NCs LED and (**c**) EL spectra of 1% P-doped Si NCs LED.

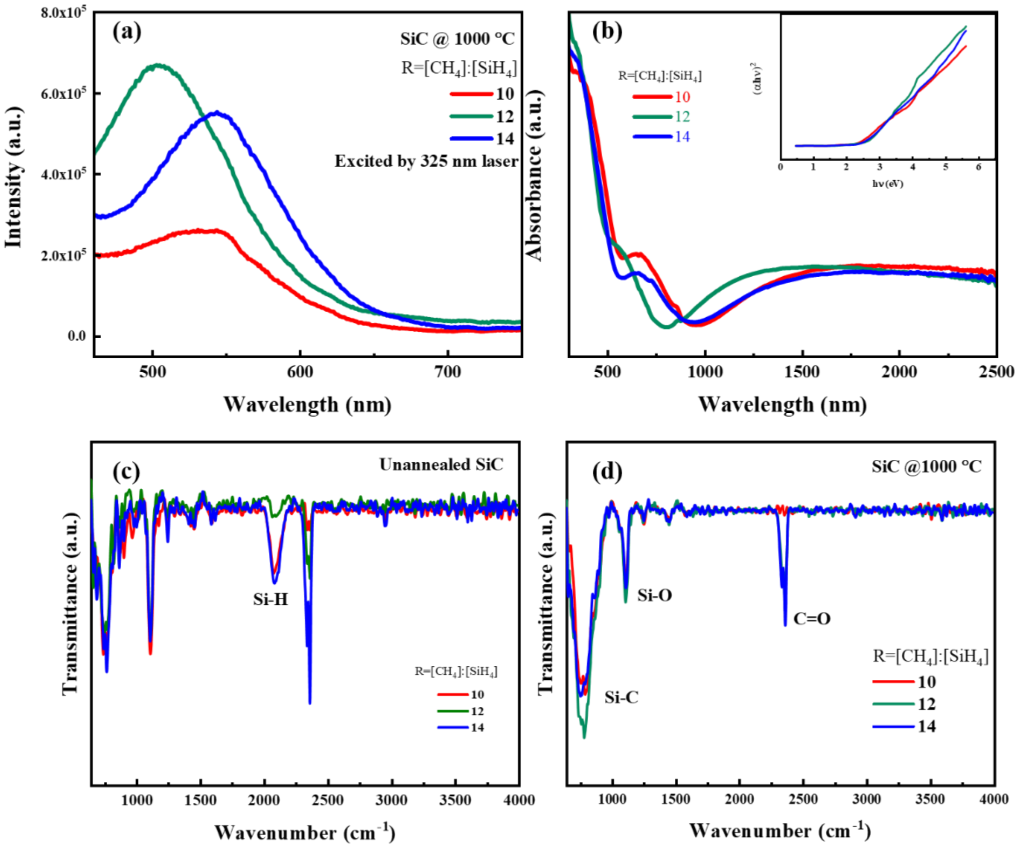

Figure S2. (**a**) PL spectrum of unannealed SiC (R = 10) excited by 325 nm laser; (**b**) Absorption spectra of annealed SiC with various R. Inset is the Tauc's plot against photon energy; FTIR spectra of unannealed (**c**) and annealed (**d**) SiC with various R.

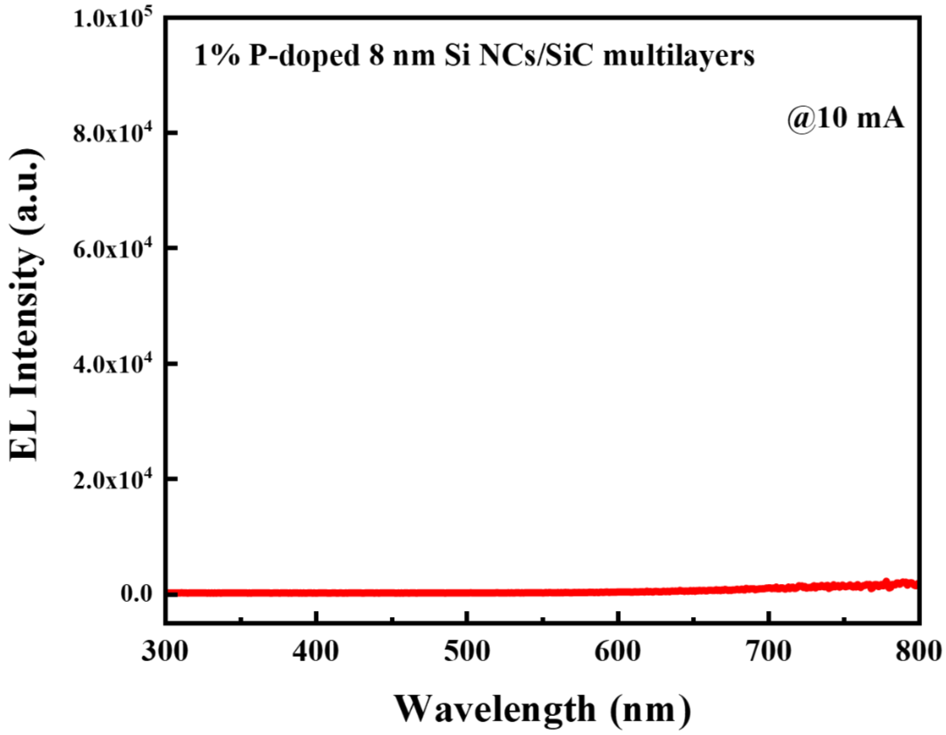

Figure S3. EL spectrum of 1% P-doped 8 nm sized Si NCs/SiC multilayers at 10 mA applied current.
